# A systematic review of outcome measures evaluating treatment efficacy in vulval lichen sclerosus and evaluation of patients' priorities

**DOI:** 10.1002/ski2.422

**Published:** 2024-07-05

**Authors:** Sara Jasionowska, Aurora Almadori, Mary Goble, Benjamin J. Langridge, Despoina Iakovou, Fady Kamel, Milla McKenzie, Allan Mclean, Deborah Boyle, Nicole Zenner, Victoria Swale, Peter E. M. Butler

**Affiliations:** ^1^ Charles Wolfson Centre for Reconstructive Surgery Royal Free Hospital London UK; ^2^ Department of Plastic Surgery Royal Free Hospital London UK; ^3^ Division of Surgery and Interventional Science University College London London UK; ^4^ Department of Obstetrics and Gynaecology Royal Free London NHS Foundation Trust Hospital London UK; ^5^ Department of Dermatology Royal Free London NHS Foundation Trust Hospital London UK

## Abstract

Vulval lichen sclerosus (VLS) is an inflammatory skin disease characterised by itching, apareunia, loss of vulval architecture and scarring. Heterogeneity in outcome reporting precludes comparison between treatments. This study aimed to systematically review outcome measures used to evaluate the efficacy of VLS treatments and present patients' treatment priorities. This review followed the PRISMA guidelines using a registered protocol (PROSPERO: CRD42022356738). Multiple databases were searched, along with grey literature on Clinicaltrials.gov, European Union Clinical Trials and International Standard Randomised controlled trial (RCT) registries. All RCTs assessing any treatment for VLS were eligible for inclusion. A total of 775 patients were assessed across 21 RCTs. The assessment tools reported outcomes in the following domains: patient‐reported symptoms assessed with one validated scale in 12 studies and seven non‐validated scales in nine studies; sexual function with validated female sexual function index and female sexual distress scale in two studies and two non‐validated scales in two studies; quality of life with three validated scales in three studies and clinician‐reported objective outcomes with two validated scales in three studies and six non‐validated scales in fourteen studies. Histological changes were assessed in 10 studies and tissue biomechanics in one study. We also carried out an online survey completed by 809 women with VLS to assess their research and disease treatment priorities and identified validated outcome measures to assess these. There is high variability in assessing treatment outcomes for VLS. We identified validated assessment tools which could be implemented in VLS studies to evaluate the effectiveness of treatments.

## Graphical abstract

Vulvar lichen sclerosus (VLS) is a chronic inflammatory skin disease affecting the anogenital areas of females. Its exact prevalence is unknown and mild cases are often unreported; the estimated prevalence in adult female patients is up to 3%. The aim of this study was to systemically review the outcome measures used to evaluate the efficacy of VLS treatments, with the goal of identifying suitable assessment tools that can be adopted in future research studies and contribute to the development of a set of core outcome measures. We also carried out an online survey completed by 809 VLS patients to assess their research and disease treatment priorities and identified validated outcome measures to assess these.

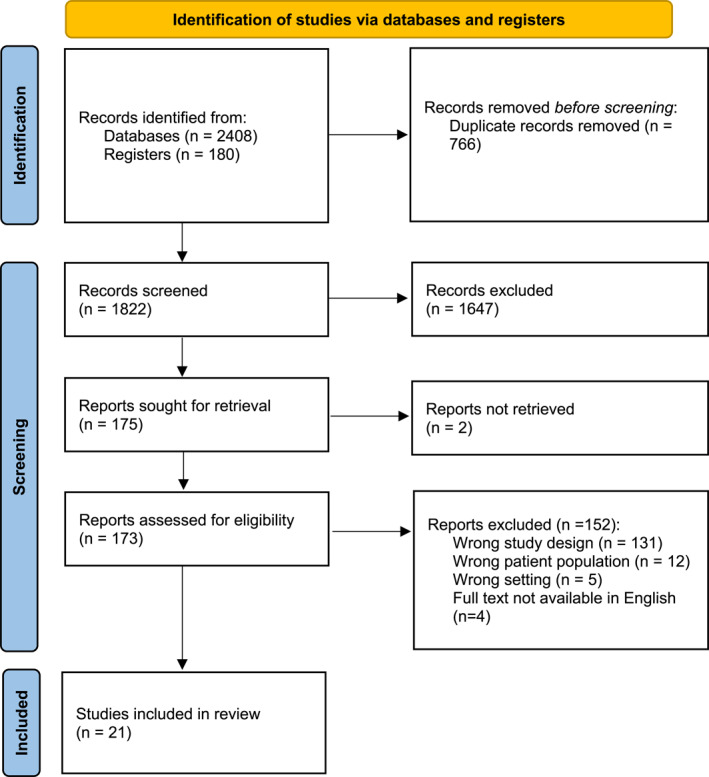

**What is already known?**

- Vulval lichen sclerosus (VLS) is a chronic skin disease characterised by debilitating symptoms negatively affecting patients' quality of life.
- The outcome assessment methods used in studies evaluating VLS treatments are too varied to allow a meaningful comparison and response to treatment.
- It is crucial to identify validated VLS assessment tools to standardise the outcome reporting and allow for the comparison of treatments.

**What does this study add?**

- Even though the outcome reporting in VLS remains heterogeneous, this systematic review identified validated VLS assessment tools which should be utilised in future studies evaluating VLS treatments.
- The identified tools assessed the patient and clinician‐reported outcomes, sexual function and quality of life.
- We present the results of the online survey completed by patients with VLS, highlighting their priorities in disease management and validated outcome measures evaluating these.

## 1 INTRODUCTION

Vulval lichen sclerosus (VLS) is a chronic inflammatory skin disease that affects the anogenital areas of females.^1^ Its exact prevalence is unknown and mild cases are often unreported; the estimated prevalence in adult female patients is up to 3%.^2^ VLS exhibits a bimodal distribution of onset, occurring predominantly in the prepubertal and post‐menopausal periods.^3^ It is one of the most common reasons for referrals for vulval distress and anatomical changes.^1^

VLS typically presents as flat, porcelain‐white spots, which may coalesce into plaques accompanied by erythema, ecchymosis, hyperkeratosis and excoriations due to itching.^1^ Commonly affected sites include the fourchette, labia minora, interlabial sulci, perineal body and clitoral hood.^2^ Inflammation‐induced scarring may lead to irreversible architectural distortion, such as labia minora fusion, vaginal introitus stenosis and clitoral burying. VLS, a clinical diagnosis and confirmatory biopsy, is not always necessary unless atypical features are present or if there is diagnostic uncertainty. Biopsy is also essential if there is any concern about neoplastic changes.^2^

VLS patients frequently experience symptoms, such as itching, burning and pain, which can be exacerbated by sexual intercourse. Erosions and fissures are the primary causes of dyspareunia, adversely impacting women's psychological well‐being and intimate relationships.^1^ The risk of developing squamous cell carcinoma in patients with untreated VLS is 3.5%–5%.^2^ Long‐term follow‐up is needed for patients with VLS to assess the disease control, response to treatment and complications.^2^

First‐line treatment during the active phase involves ultra‐potent topical corticosteroids (TCS), which have been proven to be effective, safe and inexpensive to induce remission of symptoms and stop progression to cancer.^2^ However, it is unclear how to maintain remission as there are not many studies with long‐term follow‐up. Remission of symptoms has been seen in 60%–70% of patients.^4^ Patients who have ongoing flare‐ups after 12 weeks of therapy are recommended to continue TCS as required.^5^ Other previously researched treatments include topical calcineurin inhibitors, oral retinoids, photodynamic therapy, laser therapy, PRP and autologous fat grafting.^1^ These have not been shown to be effective in high‐quality studies and some are harmful. Cases of malignancy have been reported following topical calcineurin inhibitors^6^ and oral retinoids are severely teratogenic.^7^

The outcome reporting in studies investigating treatment response in VLS is very heterogeneous which makes comparison across studies challenging.^8^ A major impediment to advancement in VLS treatment is the lack of clearly defined standardized outcome measures, or core outcome sets.^8^

This study aimed to systematically review the outcome measures used to evaluate the efficacy of VLS treatments, to identify suitable assessment tools that can be adopted in future research studies and contribute to the development of a set of core outcome measures. We also carried out an online survey completed by 809 VLS patients to assess their research studies and disease treatment priorities and identified validated outcome measures to assess these.

## 2 METHODS

This review followed the Preferred Reporting Items for Systematic Reviews and Meta‐Analysis (PRIMSA) guidelines^9^ and was registered on PROSPERO CRD42022356738.

### 2.1 Search strategy

The MEDLINE, EMBASE and Cochrane databases were searched without limitations imposed. Unpublished literature was screened on ClinicalTrials.gov, European Union Clinical Trials and the International Standard Randomised Controlled Trial Number. A departmental librarian was consulted to increase the sensitivity of the search. The search strategy included MeSH and free word terms for VLS combined using Boolean operators (Figure S1). The search was conducted from the inception of the database to 1 August 2022.

### 2.2 Eligibility criteria

The included articles had the following characteristics:

1. Female patient population with VLS.
2. Any treatment as an intervention.
3. Any outcome measure reported.
4. Randomised controlled trials (RCTs).

The excluded articles had the following characteristics:

1. Results reported jointly for different sexes.
2. Articles not reporting original research studies, for example, review articles.
3. Conference abstracts that have not been published as full‐text publications.
4. Duplicate publications, for example, articles previously published as full‐text publications.

### 2.3 Article screening

Two reviewers (DI, FK) independently screened the identified articles against the inclusion criteria, and any disputes were resolved by a third reviewer (SJ). The screening process was conducted on Covidence (Covidence, Melbourne, Australia) with titles and abstracts screened before a full‐text review.

### 2.4 Data extraction and risk of bias

Two reviewers (SJ, MG) extracted the data independently using a template in Microsoft Excel© (Microsoft, Richmond, USA). Any discrepancies were resolved by a third reviewer (AA). The risk of bias in all included studies was assessed using the Cochrane Risk of Bias 2 tool (RoB 2) for randomized controlled trials.^10^

## 3 RESULTS

**FIGURE 1:**
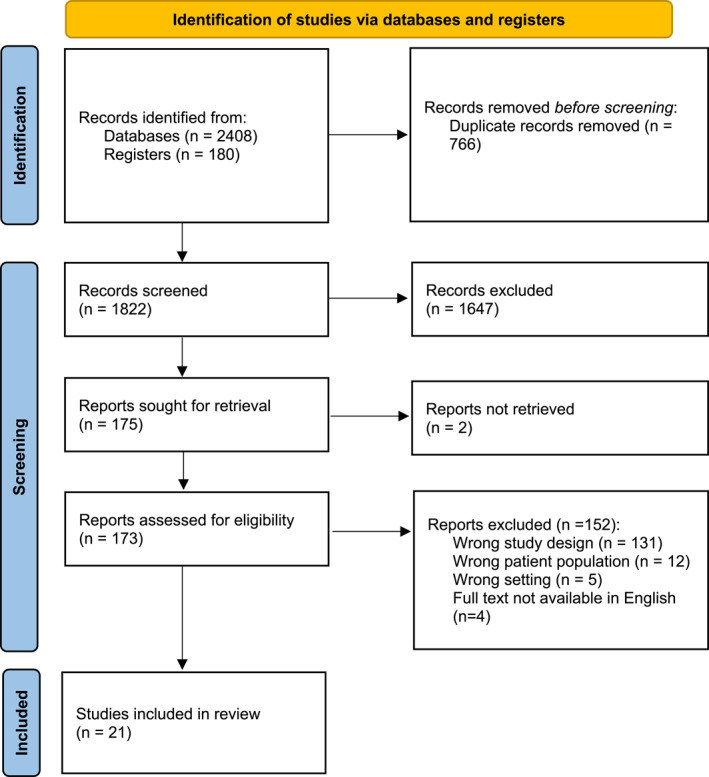
PRISMA flowchart summarising the article screening process. *Source*: From: Page MJ, McKenzie JE, Bossuyt PM, Boutron I, Hoffmann TC, Mulrow CD, et al. The PRISMA 2020 statement: an updated guideline for reporting systematic reviews. BMJ 2021;372:n71. doi: 10.1136/bmj.n71. For more information, visit: http://www.prisma‐statement.org/.

Twenty‐one RCTs met the inclusion criteria.^11^, ^12^, ^13^, ^14^, ^15^, ^16^, ^17^, ^18^, ^19^, ^20^, ^21^, ^22^, ^23^, ^24^, ^25^, ^26^, ^27^, ^28^, ^29^, ^30^, ^31^ Details of the screening process are summarised in Figure 1. There were 775 patients in total. Detailed patient demographics are shown in Table 1.

**TABLE 1:** Patient demographics in the included studies and study characteristics.

Author	Year	Study design	Total number of patients	Experimental group patient number	Control group patient number	Mean age in years ±SD (range)	Diagnostic criteria	Indication for intervention
Bijzak ogrnic^11^	2019	RCT	40	20	20	Treatment group 59 ± 10, control 57 ± 14	All histology	VLS
Borghi^12^	2015	RCT	64	32	32	Tapering group 59.4 ± 13.6, continuous 62.5 ± 11.7	Clinical diagnosis +/− histological	VLS
Burkett^13^	2021	RCT	52	27	24	Treatment group 47.3 ± 6.0, control 47.3 ± 5.8	All histology	>21 on skindex 29 score
Burrows^14^	2011	Double‐blind RCT	38	18	20	Not reported	All histology	VLS
Cattaneo^15^	1996	RCT	32	16	16	Not reported	Clinical and histology	N/A
Corazza^16^	2016	Open‐label comparative trial	48	24	24	CP group 67.33 ± 9.74, MMF group 60.96 ± 15.28	Clinical in all, 39 (72%) confirmed histologically	Patients with VLS who had previously responded to steroids on 12‐week treatment course
D'Antuono^17^	2011	Double‐blinded RCT	42	21	21	DS group 49* (IQR = 34–67 y) CT group 53 *(IQR = 44–67 y) *median age	Clinical + biopsy if uncertainty	Symptoms from 6 months to 12 years before consultation
Funaro^18^	2014	RCT	58	28	27	Tacrolimus group 46, clobetasol 45.5	All histology	Newly diagnosed or untreated for 1 month
Goldstein^19^	2011	RCT	38	17	19	Not reported	All histology	Treatment free for 4 months, score ≥4 on VAS PR/VAS BP
Goldstein^20^	2015	RCT	30			58	All histology	VLS
Goldstein^21^	2019	RCT	30	19	10	52.1	All histology	VLS
Gunthert^22^	2022	RCT	37	17	20	Progesterone 34.3 ± 10.5, clobetasol 34.7 ± 9.7	All histology	Previously untreated early onset VLS
Gutierrez‐Ontalvilla^23^	2022	RCT	20	10	10	Treatment 50.7 ± 12.1, control 59.2 ± 5.6	All clinical and histology	Symptomatic VLS refractory to topical steroid treatment
Mitchell^24^	2021	RCT, double‐blind, sham‐controlled	37	19	18	Treatment 59 (55–64), control 59 (50–65)	Clinical, histology	N/A
Origoni^25^	1996	RCT, double‐blind, cross‐over, placebo‐controlled	22	22	22	53.2 (23–71)	Clinical and histological	N/A
Paslin^26^	1991	RCT, cross‐over	5	5	5	Not reported (41–89)	Clinical and histology	N/A
Paslin^27^	1996	RCT, double‐blind cross‐over	5	5	5	Not reported (42–82)	Clinical and histology	Itching, pain, dyspareunia, architectural changes
Shi^28^	2016	RCT, open label	40	20	20	ALA group 50.7 ± 9.6, steroid group 52.1 ± 8.9	Clinical	N/A
Sideri^29^	1994	RCT, double‐blind	58	30	28	56.5 (35–83)	N/A	N/A
Virgili^30^	2014	RCT, parallel‐group, open‐label comparative trial	54	27	27	CP group 67.0 ± 9.7, MMF group 61.4 ± 14.8	Clinical in all, 39 (72%) confirmed histologically	Burning, itching, erythema, leucoderma, sclerosis scarring, hyperkeratosis, purpuric lesions and itching‐related excoriations
Virgili^31^	2013	RCT, parallel‐group, open‐label	25	8	17	Active treatment group 59.65 ± 12.30, maintenance group 60.53 ± 11.89	Clinical in all, 17 (63%) confirmed histologically	Itching, burning, dyspareunia, erythema, leucoderma, hyperkeratosis, purpuric lesions and itching‐related excoriations

### 3.1 Interventions characteristics

The included studies reported a wide variety of VLS treatments. The effectiveness and complications of all interventions are reported in Table S1.

### 3.2 Outcome measures

Assessment methods used evaluated the following aspects of VLS: patient‐reported symptoms, clinician‐reported objective outcomes, quality of life and sexual function, histological changes, tissue biomechanics and others, as presented in Table S2.

#### 3.2.1 Patient‐reported symptom severity outcome measures

All studies used assessment tools evaluating patient‐reported symptom severity. Seventy‐one percent (15/21) of studies used patient‐rated scores where symptoms such as itching, burning or dyspareunia were graded on a Likert scale ranging from 0 to 2 to 0–10 points. Twelve studies used a 0–10 visual analogue scale (VAS).^11^, ^12^, ^13^, ^16^, ^18^, ^19^, ^20^, ^21^, ^23^, ^24^, ^30^, ^31^ In 33% (7/21) of studies, the symptom‐specific scores for two to four symptoms were collated to a global symptom severity score (GSS).^12^, ^13^, ^16^, ^23^, ^24^, ^30^, ^31^

The GSS which rates four symptoms (pruritus, burning, dyspareunia and itching) from 0 (no complaints) to 10 (extreme complaints; total score range 0–40) forms the subjective component of the LS clinical scoring system (CSS).^32^ It can be used in isolation, as performed by Goldstein et al.^21^ The objective part of the LS Clinical Scoring System (CSS) comprises a physician‐administered global objective score (GOS) which rates six items (erosions, fissures, hyperkeratosis, agglutination, stenosis and atrophy) on a 3‐point Likert scale from 0 (normal findings) to 2 (severe changes; total score range 0–12). The full LS CSS was used in two studies.^22^, ^24^ Other variations of the GSS collating results for two (burning pain and pruritus) or three (burning pain, pruritus, and dyspareunia) symptoms were also reported in conjunction with a physician‐assessed GOS rating four signs (erythema, leukoderma, hyperkeratosis, purpuric lesions) on a 4‐point Likert scale (0 = normal findings to 3 = severe changes) in four studies.^12^, ^16^, ^30^, ^31^

Additionally, four studies used a 4‐point scale of symptom severity (0, no symptoms; 3, severe symptoms).^17^, ^25^, ^27^, ^28^

Funaro et al^18^ measured changes in the condition via a 6‐point scale, ranging from negative 1 (Worse) to 4 (Complete remission). Burkett et al^13^ reported patient global impression of improvement on a 0–5 point scale (0, much worse; 5, much better).

The use of a 3‐point scale graded as improved, unchanged or worsened was documented by Sideri et al.^29^

#### 3.2.2 Sexual function outcome measures

Two of the studies measured patient‐reported changes in sexual activity using non‐validated questionnaires^11^ or unspecified qualitative measures.^27^ Two studies used validated scales of sexual function.^14^, ^20^ Burrows et al^14^ used the female sexual distress scale (FSDS),^33^ a 13‐item questionnaire measuring sexually related personal distress. The female sexual function index (FSFI)^34^ which is a 19‐question 6‐domain instrument assessing sexual function, was also used by Goldstein et al.^20^

#### 3.2.3 Patient‐reported quality of life outcome measures

Three studies used validated questionnaires to assess patient quality of life.^13^, ^22^, ^23^ One study^22^ used the short form SF‐12 physical and mental health score^35^ which is a generalised health status questionnaire. One study^13^ used the Vulvovaginal symptoms questionnaire (VSQ),^36^ a 21‐item questionnaire developed from Skindex‐16, designed to measure vulvovaginal symptoms in post‐menopausal women. Each question is rated on a binary scale (0 or 1), with a score range from 0 to 20 where increasing scores indicate worse symptoms, adverse emotional impact, reduced quality of life and poorer sexual function. Skindex‐29,^37^ a validated 29‐question, 3‐domain dermatology health‐related quality of life questionnaire was used by two studies.^13^, ^23^

#### 3.2.4 Clinician‐reported outcome measures

Six studies used photography to assess changes in the vulval skin appearance.^11^, ^19^, ^20^, ^26^, ^27^, ^28^ Photographs taken before and after treatment were assessed by a clinician. Four studies used a 0–3 scale to grade the severity of lichenification, ulceration, induration,^19^, ^20^ lesion size and scale,^28^ showing significant improvement with no further details provided regarding the signs assessed.^11^ Two studies did not specify how the change in scoring was quantified through photography.^26^, ^27^

Five studies used clinical assessment of signs such as hyperkeratosis, erythema, atrophy or fissures as an outcome measure, with no further details of scale or grading.^17^, ^23^, ^25^, ^26^, ^27^

Fourteen studies used non‐validated physician‐reported outcome scores. Gutierrez‐Ontavilla et al.^23^ defined a 3‐point scale of severity of signs from 0 (normal) to 2 (severe changes). Seven studies used a 4‐point scale described as 0 (no sign/absence) to 3 (severe signs)^12^, ^16^, ^18^, ^30^, ^31^ or not described.^19^, ^20^ In nine studies, symptom‐specific scores were collated to a GOS or the investigator global assessment.^12^, ^16^, ^19^, ^20^, ^22^, ^23^, ^24^, ^30^, ^31^

Two studies used the objective clinician‐reported section of the validated CSS for VLS,^22^, ^24^ as previously described. Burkett et al^13^ used the vaginal health index (VHI)^38^ which assesses the degree of vaginal atrophy across five parameters with scores ranging from 5 to 25, with lower scores indicating greater urogenital atrophy. The same study by Burkett et al^13^ also reported using a 10‐point validated VAS; however, no further details were available.

#### 3.2.5 Histology and tissue biomechanics assessment tools

Ten studies used assessment tools evaluating histological changes in response to treatment. Two studies measured changes in elastin production,^26^, ^27^ five changes in inflammation,^11^, ^20^, ^21^, ^22^, ^24^ and one skin thickness.^11^ One study used a 7‐point scale^24^ which quantified the loss of rete pegs, the amount of dermal homogenisation, and the degree of chronic inflammation. Three studies gave no further details regarding histological assessment tools used.^14^, ^23^, ^29^ Gutierrez‐Ontavilla et al^23^ evaluated the effectiveness of lipofilling and platelet‐rich plasma versus topical clobetasol propionate and assessed the change in tissue biomechanics following treatment. Change in skin elasticity was determined using Cutometer Skin Elasticity Metre MPA 580 Dual. The clitoral hood was the target area due to its accessibility and small size with limited distribution facilitating the reproducibility of the repeated measurements.

### 3.3 Risk of bias

The risk of bias in each randomized controlled trial was evaluated using the Cochrane RoB 2 tool which evaluated bias arising from the randomisation process, deviations from intended interventions, missing outcome data, outcome measurement and selection of reported results. The risk of bias for each study is classified as low (L), some concerns (C) or high (H) (Table 2).

**TABLE 2:** Risk of bias assessment of the included studies using the Cochrane Risk of bias assessment tool 2.

	Bias arising from the randomisation process	Bias due to deviations from intended interventions	Bias due to missing outcome data	Bias in measurement of the outcome	Bias in selection of the reported result	Overall bias
Paslin 1991	H	C	L	L	C	H
Cattaneo 1996	H	H	H	H	C	H
Paslin 1996	C	C	L	L	C	C
Sideri 1994	C	C	L	L	C	C
Goldstein 2011	L	L	L	L	L	L
Origoni 1996	C	C	H	L	C	H
Burrows 2011	L	L	L	L	L	L
D'Antuono 2011	L	L	L	C	L	C
Gunthert 2022	L	L	L	L	L	L
Virgili 2014	C	C	L	C	C	C
Borghi 2015	L	L	L	L	L	L
Corazza 2016	L	L	L	C	L	C
Virgili 2013	C	C	H	C	C	H
Shi 2016	C	C	L	C	C	C
Burkett 2021	L	C	L	C	L	C
Gutierrez‐Ontalvilla 2022	C	C	L	C	C	C
Funaro 2014	L	C	L	L	L	C
Mitchell 2021	C	L	L	L	L	C
Bijzak ogrnic 2019	L	H	H	H	L	H
Goldstein 2015	L	L	L	L	L	L
Goldstein 2019	L	L	L	L	L	L

*Note*: H–High, red; L–low, green; C–some concerns, orange.

## 4 DISCUSSION

A recent meta‐analysis of treatments for VLS highlighted that a major challenge in assessing treatment response in VLS is the variability in the assessment tools used and the selection of outcome measures.^8^ This issue has been acknowledged by the Core Outcomes for Research in Lichen Sclerosus (CORALS) initiative.^39^, ^40^ The group has established the James Lind Alliance Priority Setting Partnership which highlighted the 10 future research priorities for VLS, including the identification of diagnostic criteria, assessment domains and impact on quality of life.^41^ They aimed to develop internationally accepted core outcome measures for trials addressing the treatment of vulvovaginal conditions, including VLS, which is anticipated to be published later this year.^40^ According to CORALS, the international agreement is that clinical trials for VLS should all measure signs, symptoms and quality of life as core outcomes.^40^

This systematic review aimed to analyse the assessment tools used in studies investigating any VLS treatment. This review provides an update on current trends in VLS assessment, previously reviewed by Simpson et al. in a study evaluating outcome measures for vulval skin conditions. It highlighted substantial heterogeneity in outcome reporting, with 28 studies assessing 25 outcomes using 49 different scales.^42^ Given that this review was published over a decade ago, an update of current practice is necessary.

All studies included in this systematic review employed patient‐led assessment tools. Although the tools varied between the studies, most utilised a version of a Likert scale to quantify changes in patients' symptoms. The most used tool was a VAS, ranging from 0 (no complaints) to 10 (extreme complaints), assessing itching, burning and dyspareunia. This tool has previously been described and validated for VLS by Gunthert et al.^32^ as a subjective component of the Clinical Scoring System (CSS) for VLS. The validation study included 24 patients with established VLS diagnosis and 49 patients with other vulval diseases. CSS was found to be valid, with a lack of redundancy of items (correlation coefficients <0.90) and satisfactory internal consistency (Cronbach's *α* ≥ 0.70).^32^

The assessment tools used to evaluate clinician‐rated outcomes combined Likert scales with either clinical photography or clinical assessment. Most of the tools were not validated. One validated score for VLS has been identified, a clinician‐reported three‐point Likert scale ranging from 0 (normal) to 2 (severe changes), evaluating six signs: erosions, hyperkeratosis, fissures, agglutination, stenosis, and atrophy (score range 0–12). This tool constitutes the objective part of the CSS for VLS.^32^ The score can be used to aid the diagnosis of VLS and to evaluate treatment response over time, which makes it useful both in clinical trials and in clinical practice. Furthermore, the VHI,^38^ a clinician‐rated tool specific to vaginal pathology, contrasts with other tools assessing generic skin pathology signs and symptoms. The score is used to objectively assess the degree of vaginal atrophy by measuring vaginal elasticity, pH, secretions, mucosal petechiae and hydration. However, it does not assess most of the typical VLS symptoms which are included in the CSS.

All studies reported changes in signs, including anatomical vulval changes. A joint score for the entire vulval region was reported, without distinguishing between vulval subunits. Almadori et al^43^ have highlighted that significant differences in disease severity may exist between the various vulval subunits and failure to recognise this may result in inadequate reporting of disease severity. They developed and validated the vulval architecture severity scale, a six‐region four‐point grading scale which assesses each vulval aesthetic subunit separately, making it particularly useful in a surgical intervention setting where the assessment and treatment of each vulval subunit is important.^43^

Sexual function was inadequately reported in most studies, even though VLS patients are commonly affected by dyspareunia. This can negatively impact intimate relationships, identity and self‐esteem, and can lead to avoidant behaviours, depression and anxiety.^44^ Among the included studies, only two validated scales were reported. The FSFI, which evaluates patients' feelings about various aspects of their sexual function including desire, subjective arousal, lubrication, orgasm, satisfaction and pain was validated in a study including 131 control patients and 128 age‐matched women with sexual arousal disorder and was found to have a high degree of internal consistency (Cronbach's alpha 0.82 and above) and satisfactory construct validity demonstrated by highly significant mean difference scores between the control and experimental groups.^34^ The FSDS, which assesses anxiety, guilt, frustration and distress caused by sexual dysfunction was validated by Derogatis et al. and found to have a high degree of internal consistency and test–retest reliability, with a high degree of discriminative ability to distinguish between sexually dysfunctional and functional women.^33^ These tools can be used in conjunction to comprehensively evaluate the physical and psychological aspects of patients' sexual dysfunction caused by VLS.

Quality of life, psychological well‐being, and the impact of the disease on daily activities were poorly reported. Three validated scores have been identified. Only the VSQ^36^ has been developed and validated specifically to assess vulvovaginal conditions in post‐menopausal women. However, it uses a binary (Yes/No) scale which makes its utility in addressing treatment response limited. The second validated score assessing the quality of life was Skindex‐29,^37^ which is not specific to vulval pathology. The third identified validated tool was the 12‐item short‐form health survey (SF‐12),^35^ which provides a useful overview of patients' well‐being but fails to address problems specific to VLS patients. As a result, it might be suitable as an adjunctive assessment tool rather than a stand‐alone measure of the quality of life of patients with VLS. Saunderson et al^44^ have recently validated the Vulval quality of life index (VQLI) which combines questions on symptoms severity, emotional well‐being, day‐to‐day activities, relationships, sexual intercourse and future health concerns. This short 15‐item questionnaire provides a very useful overview of the global impact of vulval disease on patients' quality of life. All identified validated VLS assessment tools have been summarised in Table 3.

**TABLE 3:** The identified validated VLS assessment tools.

Outcome type	Validated assessment tool
Patient‐reported	1. Visual analogue scale (VAS) for itching, burning and dyspareunia—the subjective part of the clinical scoring system (CSS) for VLS
Clinician‐rated	1. Likert scale (0–2) evaluating six signs, including erosions, hyperkeratosis, fissures, agglutination, stenosis, and atrophy—the objective part of the CSS for VLS
	2. Vaginal health index (VHI)
	3. Vulval architecture severity scale (VASS)
Sexual function	1. Female sexual function index (FSFI)
	2. Female sexual distress scale (FSDS)
Quality of life	1. Vulvovaginal symptoms questionnaire (VSQ)
	2. Skindex‐29
	3. 12‐Item short‐form health survey (SF‐12)
	4. Vulvar quality of life index (VQLI)

Another reported method of assessing treatment response in VLS was histological evaluation. Histological evaluation varied between the studies, and no validated assessment tool was used. Given that VLS is predominantly diagnosed clinically, the importance and benefit versus risk profile of histological assessment, performed with the use of repeated tissue biopsies to monitor treatment response, are uncertain.

One study used cutometry to assess tissue biomechanical changes in response to treatment.^23^ The Cutometer (MPA 580; Courage and Khazaka, Cologne, Germany) is a suction‐based device which measures skin elasticity using negative pressure to measure skin deformation. It has previously been used to evaluate fibrotic skin in other conditions, including the irradiated neck tissue, showing decreased skin elasticity in irradiated patients.^45^ Future studies should consider the use of the cutometer, particularly when evaluating surgical interventions aimed at reducing scarring and skin fibrosis. However, the use of the cutometer in VLS did not highlight any statistically significant differences before and after the treatment and in comparison, with the control group.^23^ Additionally, it is a suction device which may not be suitable for use in the vulval region. A validation study should be conducted and its acceptability for patients should be assessed.

Goodrum et al.^46^ published recommended outcome domains for VLS based on a survey completed by 653 respondents, including patients and healthcare professionals. The identified domains were symptoms, quality of life, sexual dysfunction, the appearance of the vulva and progression of the condition to cancer and management of the condition (improved awareness, earlier diagnosis, better treatment).

We carried out patient and public involvement work to establish what matters most to patients affected by VLS which should help define the research priorities and most adequate outcomes measures. An online survey was designed in collaboration with a clinical psychologist in our department based on the outcomes of the focus group. It was disseminated on international patient forums and VLS Facebook support groups. The survey was completed by 809 women affected by VLS. Patients were asked to rank the VLS domains identified by Goodrum et al.^46^ from 0 to 10 and the highest‐ranked signs and symptoms included changes to the anatomy of the vulva (parts of the vulva fusing together, loss of labia minora, scarring) (mean score 7.79/10), emotional impact on intimate relationships (mean score 7.65/10), inability to have penetrative intercourse (mean score 7.46/10) and pain during sexual intercourse (mean score 7.42/10). The survey participants reported that addressing the most bothersome symptoms of VLS would be life‐changing as it would allow them to perform their daily activities pain‐free, be intimate with their partner, regain the lost confidence and improve their mental health. We performed a literature search to identify further validated outcome measure tools to assess the highest‐scored domains. The results are summarised in Table 4.

**TABLE 4:** Research priorities based on an online patient survey and identified validated outcome measures.

Research priorities identified by patients	Identified validated outcome
Changes to the anatomy of the vulva (parts of the vulva fusing together, loss of labia minora, scarring)	Genital appearance assessment scale (GAAS)^47^
	Female genital self‐image scale (FGSIS)^48^
	Clitoral phimosis, interlabial sulci involvement, vulvar introitus narrowing classification (CIV)^49^
	Clinical lichen sclerosus score (CLISSCO)^50^
Emotional impact on intimate relationships	Relationship assessment scale (RAS)^51^
Inability to have penetrative intercourse	Female sexual function index (FSFI)
Pain during sexual intercourse	Pain anxiety symptom scale (PASS‐20)^52^
	Vulvar pain assessment questionnaire (VPAQ)^53^

The available published literature supporting clinical outcome assessments in VLS limits the robustness of efficacy and therefore reflects the quality of the evidence base in this paper. The included studies were limited to RCTs. The assessment tools identified were highly variable precluding meaningful quantitative meta‐analysis. Additionally, it would be unethical to carry out a placebo‐controlled RCT to produce higher‐quality evidence of VLS treatments due to known risks of worsening disease symptoms and the risk of progression to cancer. The risk of bias analysis revealed that, despite studies included being limited to RCTs, the overall risk of bias in 72% of the included studies (16/22) was ‘high’ or there were ‘some concerns’, with the bias arising mainly from the measurement of outcome and deviations from intended interventions. This highlights the concerns about the quality of the studies and their reported outcomes. This study identified validated assessment tools and the evidence base supporting their utility in line with patients' priorities and CORALS recommendations which will allow a more robust analysis of treatment efficacy. It was not designed to create new assessment tools.

This review examined the evidence behind specific outcome measures used in patients with VLS post‐intervention. The tools identified in this study should not be used in isolation or as a substitute for managing each patient's condition holistically during clinic consultation.

## 5 CONCLUSIONS

The European Commission states that VLS is a high burden under‐researched condition.^54^ Over the last decade, the evidence has been enriched by a variety of validated assessment tools which have the potential to facilitate homogenous and adequate reporting. We identified validated assessment tools which can be used in future studies to assess treatment response. A standardized VLS core outcomes set is required to appropriately evaluate the effectiveness of current and emerging treatments.

## CONFLICT OF INTEREST STATEMENT

None to declare.

## AUTHOR CONTRIBUTIONS

**Sara Jasionowska**: Conceptualisation (lead); formal analysis (lead); investigation (lead); methodology (lead); writing – original draft (lead); writing – review & editing (lead). **Aurora Almadori**: Conceptualisation (lead); data curation (equal); formal analysis (equal); methodology (lead); resources (equal); supervision (lead); writing – review & editing (equal). **Mary Goble**: Formal analysis (equal); investigation (equal); methodology (equal); writing – original draft (equal); writing – review & editing (equal). **Benjamin J. Langridge**: Conceptualisation (lead); data curation (equal); methodology (equal); project administration (equal); supervision (supporting); writing – review & editing (equal). **Despina Iakovou**: Data curation (equal); investigation (equal); methodology (equal); resources (equal); writing – original draft (equal). **Fady Kamel**: Data curation (equal); formal analysis (equal); methodology (equal); writing – original draft (equal). **Milla McKenzie**: Formal analysis (equal); methodology (equal); resources (equal); writing – original draft (equal). **Allan MacLean**: Conceptualisation (equal); methodology (equal); supervision (equal); writing – review & editing (equal). **Deborah Boyle**: Conceptualisation (equal); methodology (equal); supervision (equal); writing – review & editing (equal). **Nicole Zenner**: Conceptualisation (equal); methodology (equal); supervision (equal); writing – review & editing (equal). **Victoria Swale**: Conceptualisation (equal); methodology (equal); supervision (equal); writing – review & editing (equal). **Peter E. M. Butler**: Conceptualisation (lead); methodology (lead); supervision (lead); writing – review & editing (lead).

## ETHICS STATEMENT

Not applicable.

## Supporting information

Supporting Information S1

Figure S1

Table S1

Table S2

Table S3

## Data Availability

All data collected during this study will be included in the published article.
