# Supplementary material for: A systematic review of outcome measures evaluating treatment efficacy in vulval lichen sclerosus and evaluation of patients' priorities
Source: Skin Health Dis. 2024 Jul 5;4(5):e422. doi: 10.1002/ski2.422 (PMC11442075; doi:10.1002/ski2.422)
Supplement: Supplementary file 1 — Supporting Information S1 [file SKI2-4-e422-s002.docx]

Supplementary Figure 1 – The systematic search strategy.

Table 1 – Patient demographics in the included studies and study characteristics.

| **Author** | **Year** | **Study design** | **Total numberof patients** | **Experimental group patient number** | **Control group patient number** | **Mean age in years ±SD (range)** | **Diagnostic criteria** | **Indication for intervention** |
| --- | --- | --- | --- | --- | --- | --- | --- | --- |
| Bijzak Ogrnic^11^ | 2019 | RCT | 40 | 20 | 20 | Treatment group 59 ±10, Control 57±14 | All histology | VLS |
| Borghi^12^ | 2015 | RCT | 64 | 32 | 32 | Tapering group 59.4±13.6, Continuous 62.5±11.7 | Clinical diagnosis +/- histological | VLS |
| Burkett^13^ | 2021 | RCT | 52 | 27 | 24 | Treatment group 47.3±6.0, Control 47.3±5.8 | All histology | >21 on Skindex 29 score |
| Burrows^14^ | 2011 | Double-blind RCT | 38 | 18 | 20 | Not reported | All histology | VLS |
| Cattaneo^15^ | 1996 | RCT | 32 | 16 | 16 | Not reported | Clinical and histology | N/A |
| Corazza^16^ | 2016 | Open-label comparative trial | 48 | 24 | 24 | CP group 67.33±9.74, MMF group 60.96±15.28 | Clinical in all, 39 (72%) confirmed histologically | Patients with VLS who had previously responded to steroids on 12-week treatment course |
| D'Antuono^17^ | 2011 | Double-blinded RCT | 42 | 21 | 21 | DS group 49* (IQR = 34-67 y) CT group 53*(IQR = 44-67 y) *Median age | Clinical + biopsy if uncertainty | Symptoms from 6months to 12 years before consultation |
| Funaro^18^ | 2014 | RCT | 58 | 28 | 27 | Tacrolimus group 46, Clobetasol 45.5 | All histology | Newly diagnosed or untreated for 1 month |
| Goldstein^19^ | 2011 | RCT | 38 | 17 | 19 | Not reported | All histology | Treatment free for 4 months, score >=4 on VAS PR/VAS BP |
| Goldstein^20^ | 2015 | RCT | 30 |  |  | 58 | All histology | VLS |
| Goldstein^21^ | 2019 | RCT | 30 | 19 | 10 | 52.1 | All histology | VLS |
| Gunthert^22^ | 2022 | RCT | 37 | 17 | 20 | Progesterone 34.3±10.5, Clobetasol 34.7±9.7 | All histology | Previously untreated early onset VLS |
| Gutierrez-Ontalvilla^23^ | 2022 | RCT | 20 | 10 | 10 | Treatment 50.7±12.1, Control 59.2±5.6 | All clinical and histology | Symptomatic VLS refractory to topical steroid treatment |
| Mitchell^24^ | 2021 | RCT, double blind, sham-controlled | 37 | 19 | 18 | Treatment 59 (55-64), Control 59 (50-65) | Clinical, histology | N/A |
| Origoni^25^ | 1996 | RCT, double-blind, cross-over, placebo-controlled | 22 | 22 | 22 | 53.2 (23-71) | Clinical and histological | N/A |
| Paslin^26^ | 1991 | RCT, cross-over | 5 | 5 | 5 | Not reported (41-89) | Clinical and histology | N/A |
| Paslin^27^ | 1996 | RCT, double-blind cross-over | 5 | 5 | 5 | Not reported (42-82) | Clinical and histology | Itching, pain, dyspareunia, architectural changes |
| Shi^28^ | 2016 | RCT, open label | 40 | 20 | 20 | ALA group 50.7±9.6, Steroid group 52.1±8.9 | Clinical | N/A |
| Sideri^29^ | 1994 | RCT, double-blind | 58 | 30 | 28 | 56.5 (35-83) | N/A | N/A |
| Virgili^30^ | 2014 | RCT, parallel-group, open-label comparative trial | 54 | 27 | 27 | CP group 67.0±9.7, MMF group 61.4±14.8 | Clinical in all, 39 (72%) confirmed histologically | Burning, itching, erythema, leucoderma, sclerosis scarring, hyperkeratosis, purpuric lesions and itching-related excoriations |
| Virgili^31^ | 2013 | RCT, parallel-group, open-label | 25 | 8 | 17 | Active treatment group 59.65 ±12.30, Maintenance group 60.53±11.89 | Clinical in all, 17 (63%) confirmed histologically | Itching, burning, dyspareunia, erythema, leucoderma, hyperkeratosis, purpuric lesions and itching-related excoriations |

Table 2 – Risk of bias assessment of the included studies using the Cochrane Risk of bias assessment tool 2 (H- high, L – low, C- some concerns).

|  | **Bias arising from the randomisation process** | **Bias due to deviations from intended interventions** | **Bias due to missing outcome data** | **Bias in measurement of the outcome** | **Bias in selection of the reported result** | **Overall bias** |
| --- | --- | --- | --- | --- | --- | --- |
| Paslin 1991 | H | C | L | L | C | H |
| Cattaneo 1996 | H | H | H | H | C | H |
| Paslin 1996 | C | C | L | L | C | C |
| Sideri 1994 | C | C | L | L | C | C |
| Goldstein 2011 | L | L | L | L | L | L |
| Origoni 1996 | C | C | H | L | C | H |
| Burrows 2011 | L | L | L | L | L | L |
| D'Antuono 2011 | L | L | L | C | L | C |
| Gunthert 2022 | L | L | L | L | L | L |
| Virgili 2014 | C | C | L | C | C | C |
| Borghi 2015 | L | L | L | L | L | L |
| Corazza 2016 | L | L | L | C | L | C |
| Virgili 2013 | C | C | H | C | C | H |
| Shi 2016 | C | C | L | C | C | C |
| Burkett 2021 | L | C | L | C | L | C |
| Gutierrez-Ontalvilla 2022 | C | C | L | C | C | C |
| Funaro 2014 | L | C | L | L | L | C |
| Mitchell 2021 | C | L | L | L | L | C |
| Bijzak Ogrnic 2019 | L | H | H | H | L | H |
| Goldstein 2015 | L | L | L | L | L | L |
| Goldstein 2019 | L | L | L | L | L | L |

Table 3 – The identified validated VLS assessment tools.

| Outcome type | Validated assessment tool |
| --- | --- |
| Patient-reported | 1. Visual Analogue Scale (VAS) for itching, burning and dyspareunia – the subjective part of the Clinical Scoring System (CSS) for VLS |
| Clinician-rated | 1. Likert scale (0-2) evaluating six signs, including erosions, hyperkeratosis, fissures, agglutination, stenosis, and atrophy - the objective part of the CSS for VLS 2. Vaginal Health Index (VHI) 3. Vulvar Architecture Severity Scale (VASS) |
| Sexual function | 1. Female Sexual Function Index (FSFI) 2. Female Sexual Distress Scale (FSDS) |
| Quality of life | 1. Vulvovaginal Symptoms questionnaire (VSQ) 2. Skindex-29 3. 12-item short-form health survey (SF-12) 4. Vulvar quality of life index (VQLI) |

Table 4. Research priorities based on an onlne patient survey and identified validated outcome measures.

| Research priorities identified by patients | Identifed validated outcome |
| --- | --- |
| Changes to the anatomy of the vulva (parts of the vulva fusing together, loss of labia minora, scarring) | Genital Appearance Assessment Scale (GAAS)^47^  Female Genital Self-Image Scale (FGSIS)^48^  Clitoral phimosis, interlatial sulci involvement, vulvar introitus narrowing classification (CIV)^49^ |
| Emotional impact on intimate relationships | Relationship Assessment Scale (RAS)^50^ |
| inability to have penetrative intercourse | Female Sexual Function Index (FSFI) |
| Pain during sexual intercourse | Pain Anxiety Symptom Scale (PASS-20)^51^  Vulvar pain Assessment Questionnaire (VPAQ)^52^ |

Supplementary table legends

Supplementary Table 1 – The effectiveness of all VLS treatments reported in the included studies.

Supplementary Table 2 - Table 2: Outcome Measures Reported in the included studies.

Supplementary Table 3 – The PRISMA checklist.
