# Supplementary material for: A systematic review of outcome measures evaluating treatment efficacy in vulval lichen sclerosus and evaluation of patients' priorities
Source: Skin Health Dis. 2024 Jul 5;4(5):e422. doi: 10.1002/ski2.422 (PMC11442075; doi:10.1002/ski2.422)
Supplement: Supplementary file 2 — Figure S1 [file SKI2-4-e422-s001.docx]

Supplementary Figure 1

| Embase |
| --- |
| 1 vulva kraurosis/ |
| 2 Lichen Sclerosus et Atrophicus/ |
| 3 Vulva/ |
| 4 2 and 3 |
| 5 1 or 4 |
| 6 Vulval lichen sclerosus or vulval LS or vulval sclerosis or Vulval lichen sclerosus or VLS or kraurosis vulva* or lichen sclerosus of vulva* |
| 7 Vulva or vulval or vulva* or clitoris or labia |
| 8 Lichen sclerosus or lichen sclerosis |
| 9 7 and 8 |
| 10 5 or 6 or 9 |
| 11 random* or control* or RCT |
| 12 10 and 11 |

| Medline |
| --- |
| 1. Vulvar Lichen Sclerosus/ |
| 2. Lichen Sclerosus et Atrophicus/ |
| 3. Vulva/ |
| 4. 2 and 3 |
| 5. 1 or 4 |
| 6. Vulval lichen sclerosus or vulval LS or vulval sclerosis or Vulval lichen sclerosus or VLS or kraurosis vulva* or lichen sclerosus of vulva* |
| 7. Vulva or vulval or vulva* or clitoris or labia |
| 8. Lichen sclerosus or lichen sclerosis |
| 9. 7 and 8 |
| 10. 5 or 6 or 9 |
| 11. random* or control* or RCT |
| 12. 10 and 11 |

| Cochrane Library |
| --- |
| Vulvar lichen sclerosus AND random* or control* or RCT |
