# Supplementary material for: A systematic review of outcome measures evaluating treatment efficacy in vulval lichen sclerosus and evaluation of patients' priorities
Source: Skin Health Dis. 2024 Jul 5;4(5):e422. doi: 10.1002/ski2.422 (PMC11442075; doi:10.1002/ski2.422)
Supplement: Supplementary file 3 — Table S1 [file SKI2-4-e422-s003.docx]

| **Author and Year**  Supplementary Table 1: The effectiveness of all VLS treatments reported in the included studies. | **Therapeutic and control interventions** | **Intervention details** | **Concomitant steroid therapy** | **Patient rated outcomes: Symptoms** | **Patient rated QOL outcomes** | **Physician rated sign-based outcomes** | **Objective: fibrosis** | **Objective: histology** | **Complications** | **Follow-up duration** | **Adherence** |
| --- | --- | --- | --- | --- | --- | --- | --- | --- | --- | --- | --- |
| Paslin 1991 | Topical dihydrotestosterone 2% vs petrolatum ointment | Each topical treatment was applied twice a day every day for 6 months. | Other androgen or corticosteroid-based treatments stopped 1 month before the start of the trial. | No improvement in itching or dyspareunia in both groups. | N/A | Increase in clitoral size in 4/5 (80%) of in experimental group which reversed upon treatment cessation. No improvement in the control group. 5/5 (100%) patients had an objective improvement of lichen features, assessed by photography. 4/5 (80%) patients showed increased elastic fibres production on dihydrotestosterone therapy, no change was seen in the control group. | N/A | Histological evaluation of a change in elastin production. | N/A | 6 months | N/A |
| Cattaneo 1996 | Topical testosterone propionate 2% vs petrolatum ointment | Each topical treatment was applied once a day for 24 weeks. | N/A | Testosterone group: worsening of symptoms, especially itching in 9/16 (56.3%). Control group: 3/16 (18.8%) reported soothing effect, 3/16 (18.8%) itching recurrence. Better repones reported for placebo but no details provided. | N/A | N/A | N/A | N/A | 4/16 (25%) in experimental group experienced burning | 24 weeks | N/A |
| Paslin 1996 | Topical dihydrotestosterone 2% vs topical testosterone propionate 2% | Each topical treatment was applied twice a day every day for 6 months. | Patients were asked to stop any other androgen or corticosteroid-based treatments 1 month before the start of the trial. | Both treatments alleviated pain and facilitated fissure healing but failed to improve the itching in 4/5 patients (80%). 2/5 patients were sexually active and 1/2 (50%) reported resolution of dyspareunia and ability to orgasm after treatment and 1/2 (50%) reported ongoing dyspareunia, bleeding, and inability to orgasm. | N/A | All patients had improved vulval skin appearance, 4/5 (80%) of patients showed marginally greater improvement on Testosterone therapy. Observed changes included diminished hyperkeratosis and fissures. | N/A | No difference in the capacity of both treatments to form elastic fibres or composition of stratum spinosum and basal cell layer or melanocytes. | N/A | 6 months | N/A |
| Sideri 1994  Table 3 (continued): Efficacy of Vulval Lichen Sclerosus Treatment | Topical testosterone propionate 2% vs petrolatum ointment | 2% testosterone propionate OR petrolatum ointment three times a day for 2 months, then twice a day for 10 months | N/A | Testosterone group: 20/30 (66.6%) improved, 6/30 (20.0%) unchanged, 4/30 (13.4%) worsened. Control group: 21/28 (75%) Improved, 5/28 (17.8%) unchanged, 2/28 (7.1%) worsened. No statistically significant difference between treatments. | N/A | N/A | N/A | No histological changes compared to baseline in either group. | Testosterone group: 4/30 (13.4%) patients had to stop treatment due to side effects 1/30 (3.33%) - facial hypertrichosis, 3/30 (10%) worsening pruritus and pain. | 1 year, assessed at 2-monthly intervals | N/A |
| Goldstein 2011 | Topical pimecrolimus 1% vs topical clobetasol propionate 0.05% | Pimecrolimus topical 1% BD application for 12 weeks OR clobetasol propionate 0.05% topical ON and blank cream OM for 12 weeks. | N/A | Mean change in VAS PR: clobetasol 4.5, pimecrolimus 3.5, p=0.2. Mean Change in VAS BP clobetasol 3.7 vs 3.8 pimecrolimus p=0.9 | N/A | Investigator Global Assessment (IGA) decreased compared to baseline in both groups, no comparison between groups reported. | N/A | Biopsy samples evaluated by dermatopathologist to evaluate the change in inflammation. | No adverse events, no safety concerns | 12 weeks after the start of treatment | All completed trial |
| Origoni 1996 | Topical oxatomide gel 5% vs petrolatum ointment | Topical treatment for 14 days followed by a wash-out period of 7 days when only placebo (petrolatum ointment) was administered. Each patient was then allocated to the opposite group to repeat this cycle (cross-over trial). | No other topical or systemic drug was used during the study period. | Oxamide group: Itching improved in 19/19 patients (100%) to moderate or absent. 26% of patients had complete resolution of pruritus compared to 5.3% of control group patients. There was no difference in burning or dyspareunia between both groups. | N/A | Results not reported. | N/A | N/A | 3/22 (13.6%) severe burning resulting in dropping out of the study. | 5 weeks | N/A |
| Burrows 2011 | Topical pimecrolimus 1% vs topical clobetasol propionate 0.05% | Topical twice daily application of pimecrolimus 1% for 12 weeks OR topical clobetasol 0.05% ON, unmedicated cream OM for 12 weeks. | None | N/A | Female Sexual Distress Scale (FSDS): Clobetasol pre-treatment score 29 and post-treatment 15, p=0.001. Pimecrolimus pre-treatment=27 vs post-treatment =21 p=0.001. Clobetasol improved more than pimecrolimus p=0.003. | N/A | N/A | Not reported. | N/A | 12 weeks | All completed trial |
| D'Antuono 2011 | Dermasilk briefs vs cotton underwear | Daily use of Dermasilk briefs OR cotton briefs. | Clobetasol proprionate 0.05% topical fingertip (0.25g) ON + vitamin E cream OM for 6 months | Burning before silk briefs use 21/21, after 9/21; before cotton briefs use 21/21 after 21/21, p<0.0001. Soreness before silk briefs use 21/21, after silk briefs use 0/21; before cotton briefs use 21/21, after 17/21 p<0.0001. | N/A | Silk briefs group erythema before 21/21, after 9/21 versus cotton briefs before 21/21, after 19/21 (p<0.05), all others no significant change. | N/A | N/A | No adverse effects reported. 4 patients developed candida vulvovaginitis | 1 month and 6 months after start of trial | Adherence (self-reported) 100%. |
| Gunthert 2022 | Topical progesterone cream 8% vs topical clobetasol proprionate 0.05% | 2g of progesterone cream BD for 12 weeks OR 2g of clobetasol propionate 0.05% BD for 12 weeks. | N/A | Symptom severity score change (SF-12 physical and mental health score and patient symptom score of pruritus, itching, burning) 4.5 (SD 3.8) to 3.1 (SD 3.0) in the progesterone arm, and from 4.7 (SD 2.8) to 1.9 (SD 1.8) in the clobetasol propionate arm (difference in favour of clobetasol 1.32; 95% CI − 0.25 to 2.89, p = 0.095). | N/A | Mean Clinical severity score for VLS change at 12 weeks, 4.6 (SD 2.0) to 4.5 (SD 1.7) in the progesterone arm, and from 4.6 (SD 2.8) to 2.9 (SD 2.2) in the clobetasol propionate arm (difference in favour of clobetasol 1.61; 95% CI 0.44 to 2.77, p = 0.009). | N/A | VLS in remission in 6/10 patients (60%) with available biopsy in the progesterone arm, and in 13/16 patients (81.3%) in the clobetasol arm (odds ratio in favour of clobetasol 0.35; 95% CI 0.06 to 2.06, p = 0.234). | Similar adverse events in progesterone vs control. Severe adverse event 12% progesterone vs 0% clobetasol (skin irritation /vulvitis). | 12 weeks | N/A |
| Virgili 2014  Table 3 (continued): Efficacy of Vulval Lichen Sclerosus Treatment | Topical clobetasol propionate ointment 0.05% vs topical mometasone furoate 0.1% ointment | 12-week reducing regimen of both topical agents; initially once a day for 5 days/a week for 4 weeks, then on alternate days for 4 weeks and for the third month, twice weekly. | No additional steroids or immunosuppressive treatments were used. | ≥ 75% improvement in Global Subjective Score (GSS): Clobetasol group 16/27 (59%), Mometasone group 18/27(67%). ≥ 50% improvement in GSS: Clobetasol group 21/27(78%), Mometasone group 21/27 (78%). No statistical difference between groups in both outcomes. | N/A | ≥ 75% improvement in Global Objective Score (GOS): CP group 10/27 (37%), MMF group 13/27 (48%). ≥ 50% improvement in GOS: Clobetasol group 21/27(78%), Mometasone group 22/27 (82%). No statistical difference between groups in both outcomes. | N/A | N/A | No side effects  2/27 (8%) of Clobetasol group patients were dissatisfied with treatment, as were 4/27 (15%) in the Mometasone group. | 12 weeks | All patients were adherent. |
| Borghi 2015 | Topical mometasone furoate 0.1% continuous application vs topical mometasone furoate 0.1% tapering dose | Continuous application of 0.1% Mometasone Furoate for 4 weeks, for 5 days each week (experimental) OR topical Mometasone Furoate 4 weeks OD on five days a week, every other day 4 weeks, twice weekly 4 weeks (control). | N/A | ≥75% improvement from baseline GSS: Experimental 62% versus 69% in control group. ≥50% improvement from baseline GSS: Experimental 69%% versus 72% in control group. No significant difference between groups. | N/A | ≥75% improvement from baseline GOS: Experimental 28% versus 47% in control group. ≥50% improvement from baseline GOS: Experimental 66% versus 75% in control group. No significant difference between groups. | N/A | N/A | Early return to VLS unit for symptom exacerbation - none returned. | 12 weeks after the start of treatment | Adherence: relative risk of adherence in Rx vs control group = 2.14, p=0.6 Not significant. |
| Corazza 2016 | Topical mometasone furoate 0.1% vs topical clobetasol proprionate 0.05% | Twice weekly mometasone fumarate 0.1% for 52-week maintenance phase OR topical clobetasol 0.05% twice weekly for 52 weeks maintenance phase. | N/A | GSS pre-clobetasol treatment =12, post=10. GSS pre-mometasone treatment=7, post=10. No significant difference. | N/A | GOS pre-treatment with Clobetasol=20, post=13. GOS pre-treatment with Mometasone=18, post MMF=11. No significant difference. | N/A | N/A | No adverse events.  Clobetasol group 8.33% relapse, Mometasone group 4.17%. Convenience of treatment protocol - 6.82% found inconvenient, significant association with relapse p=0.008. | 12-week intervals for 52 weeks | 40/44(90.91%) were adherent to maintenance therapy while 4 (2 in CP group and 2 in MMF group) were not. |
| Virgili 2013 | Topical mometasone furoate 0.1% vs topical vitamin E cream or cold cream | Mometasone furoate 0.1% ointment twice weekly OR Cold cream once a day or topical vitamin E once a day. | Only topical steroids, no oral steroids were allowed | N/A | N/ | N/A | N/A | N/A | No side effects  Mometasone group relapse: 0/27 (0%), Cold cream group 5/8 (62%), vitamin E group 5/9 (56%). Significantly higher in both vitamin E (p=0.0204) and cold cream (p=0.0128) groups compared to Mometasone (OR = 0.0951 95%CI 0.0177-1.5106). The median time to relapse was 21.6 weeks for patients in both vitamin E and col cream groups. 8/10 (80%) relapsed occurred during the first 6 months. | 52 weeks | 20/24 (83%) of patients who completed the study protocol were adherent. |
| Shi 2016 | 5-Aminolevulinic Acid Photodynamic Therapy vs Topical Clobetasol Propionate 0.05% | 5-aminolevulinic photodynamic therapy (ALA-PDT) four sessions administered at 2-week intervals OR clobetasol proprionate 0.05% daily for 8 weeks | No other therapeutic agents were allowed during the treatment and follow-up. | ALA-PDT: 14/20 (70%) achieved complete response, 4/20 (20%) partial response, 2/20 (10%) minimal response. Clobetasol propionate: 7/20 (35%) complete response, 6/20 (30%) partial response, 7/20 (35%) minimal response. The rate of complete response was significantly higher in the ALA-PDT group. Relapse seen in 1/14 (7.1%) of ALA-PDT group and in clobetasol propionate group 7/7 (100%) of patients relapsed at 6 months. | N/A | Both groups showed a significant reduction in lesion size but there was no difference between the 2 groups (p=0.116). | N/A | N/A | ALA-PDT: 6/20 (30%) redness and swelling, 1/20 (5%) erosion (treated with mupirocin ointment) | Patients were evaluated at 2,4,6 and 8 weeks after the start of treatment and 6 months after the end of treatment. | N/A |
| Burkett 2021 | Fractionated Carbon Dioxide Laser vs topical clobetasol propionate 0.05% | Three laser treatments 4-6 weeks apart OR clobetasol proprionate 0.05%for topical use nightly for 1 month, 3 times /week for 2 months, then as required. | Washout period for 8 weeks before enrolment if using steroids/immunomodulator. | PGI-I: 89% (23/27) of patients in the laser group rated symptoms as being “better or much better” compared with 62% (13/24) of patients in the steroid group, p=0.07. VSQ secondary patient symptom outcome mean score changed from baseline to 6 months, with a significant difference in laser (-3.93 +/- 4.12) compared with steroid (-0.58+/- 5.11; P=0.014), with more negative scores indicating greater improvement. VAS significantly more improved irritation and tearing in laser group compared to clobetasol (-4.15 vs -1.32, p=0.09, treatment effect 2.79). | Skindex-29 score at 6 months. Laser -16.83 (+-18.09) vs steroid -5.92(+-5.81); P=0.007. Subsets include emotion, function and symptoms. Emotion (laser -19.63+/-21.92 vs steroid -6.77+/-9.9; P=.011) and symptoms (laser -21.03+/-22.18 vs steroid-4.91+/-11.19; P=.002); function sub score was similar between groups (laser-10.65+/-18.97 vs steroid -5.30+/-8.64; =5.210). When stratified for previous steroid exposure, only previously exposed group had improvement in laser -21.96(+-20.25) vs steroid-6.10 (+/-4.95) p= .004 | Mean change of VHI score between baseline and 6 months in laser (1.92+/-4.34) compared with steroid (0.43+/-3.62; P=0.046), lower score corresponding to greater urogenital atrophy. | N/A | N/A | Treatment group: burning, irritation and poor healing at laser treatment site, n=1. Control group: reactivation of genital herpes, n=1. One participant started oral steroids (deviation from protocol). N=3 laser and n=1 steroid started oestrogen therapy after baseline but discontinued before 6 month follow up. | 2 weeks phone check post-enrolment, optional 3-month follow-up, 6 month follow-up, 12 month follow-up. | n=1 dropout in steroid group |
| Gutierrez-Ontalvilla 2022 | Lipofilling and Platelet-Rich Plasma vs topical clobetasol propionate 0.05% | Nanofat-PRP infiltration two sessions separated by 3 months OR topical 0.05% Clobetasol propionate twice weekly | N/A | Experimental group showed significant improvement in clinical symptoms scores ((itching, P = 0.039; pain, P = 0.016, burning, P = 0.002, dyspareunia, P = 0.035, and GSS, P = 0.006), while no significant improvement was observed in the Control group (itching, P = 0.033939; pain, P = 0.250, burning, P = 0.477, dyspareunia, P = 0.453, and GSS, P = 0.469). | Patient QoL improved from baseline in experimental group (79.7 ± 33.2 to 59.7 ± 24.9, P = 0.004). Patients in the treatment group had higher QoL scores compared to the control group (P = 0.006). | Change in clinical signs: fissures (P = 0.017), stenosis (P = 0.043), pallor (P = 0.004), and the GOS scores (P = 0.004) significantly improved in the treatment group compared to baseline. No improvement was documented in terms of the hyperkeratosis, agglutination, or atrophy scores (P = 0.500), P = 0.250; and P = 0.500, respectively). Patients from the control group did not show significant improvements from the baseline condition (all P > 0.05). At the 1-year follow-up, treatment with nanofat–PRP had proven to be significantly more effective compared to control treatment in reducing the erosions, fissures, stenosis, and pallor caused by VLS (all P > 0.05). | The mean relative values of gross and net elasticity did not improve from baseline. No significant differences between the groups were observed in elasticity at 12-month follow-up. | Histologically, treatment group showed a significant decrease in the number of inflammatory cells (all P < 0.05) , while in the control group, only the number of eosinophils diminished (P = 0.016). | No treatment related adverse events. | 1,3,6,12 months after the baseline visit for the control group or after the first infiltration of nanofat-PRP for the treatment group |  |
| Funaro 2014 | Topical tacrolimus 0.1% vs topical clobetasol propionate 0.05% | Topical tacrolimus 0.1% ON for 3 months OR clobetasol propionate 0.05% topical ON for 3 months | N/A | No difference on VAS PR and VAS BP between groups. | N/A | Clobetasol group had greater decrease in atrophic patches in perineal and clitoral area compared to tacrolimus (p=0.036+0=0.001). End of study 9 patients in Clobetasol vs 19 in tacrolimus group still had signs p=0. 015.Number of patients with no signs/symptoms in Clobetasol group 15 vs 4 tacrolimus at end of study p=0.002. | N/A | N/A | At least 1 side effect: n=20 clobetasol group, n= 24 tacrolimus group (x2 = 2.27, P = .207). Tacrolimus reported more burning sensation compared with the clobetasol group (22 vs 13: x2 = 6.84, P = .014). Both groups (tacrolimus n=3, clobetasol n=2) had patients with side effects which required reducing dose. | Monthly follow up (initial, then x3 visits) | All adherent |
| Mitchell 2021 | Fractionated Carbon Dioxide Laser vs sham laser | Fractionated CO2 laser treatment or sham laser, 5 sessions over a 24-week period at 4-weekly intervals | No, all topical and systemic treatments were stopped 4 weeks before the start of the trial. | Patient-reported CSS change: 7.10-point reduction (improvement) in the experimental group and 4.80-point reduction in the sham treatment group (difference not statistically significant). | N/A | Clinician-assessed CSS change: 0.70 increase (worsening) in the experimental group and a 0.30 reduction in the sham treatment group (difference not statistically significant). | N/A | 0.20 reduction (improvement) in histopathology scale score from baseline in the experimental group and 0.10 in the control group (95% CI -1.14,1.06, p=0.76). | N/A | 30 weeks | All patients were adherent. |
| Bijzak Ogrnic 2019 | Nd:YAG laser 1064nm vs topical betamethasone | 3 laser treatments in total every 14 days OR topical betamethasone was applied one week in decreasing regimen before first laser session first week, OD second week, every second day on third week. | All patients discontinued hormone therapy and other topical steroid therapy before trial. | VAS score sum of burning, itching and pain at 1month df=1,32, effect size 8.4 (3.8–13.1) p=0.001. At 3 months df=1,33, effect size 10.2 (6.5–14.0) p<0.001. At 6 months df=1,16, effect size7.6 (-1.0–16.1) p=0.080 | Patient sexual dysfunction decreased from baseline in laser group at 3 months, not maintained at 6 months. 12/20, 11/20, 11/20, and 7/16 patients in the laser group were sexually active at baseline and at 1-, 3-, and 6-month follow-up, respectively. 10/18, 9/15, 11/16, and 1/3 patients in the control group were sexually active at baseline and at 1-, 3-, and 6-month follow-up. | Evaluation of photographs at baseline vs 3 months post-treatment: classify before/after + give improvement score 1-4. At 3 months, able to tell difference between before/after photos in 15/20 laser and 4/11 control, chi-square= 4.47; P = 0.035. No difference in mean improvement score between groups 0.58 +/- 1.25 in the laser group and -0.27 +/-1.27 in the control group (F1,29 = 3.28; P = 0.80; effect size 0.856 +/- 0.472; 95% CI -0.110 to 1.822) | N/A | Vulva punch biopsies: at 3 months, n=9/17 laser and 6/16 control negative for lichen (no sig difference); the thickness of sclerosis reduced significantly after laser treatment (-0.67 mm; 95% CI -0.99 to -0.34 mm; P = 0.009) but not after corticosteroid treatment (-0.1 mm; 95% CI -0.48 to 0.20 mm; P = 0.577). The improvement was statistically significantly better in the laser group (by 0.57 mm; 95% CI 0.01−1.13 mm; P = 0.46). | No reported adverse effects in laser group  Laser group only - tolerability of laser: minimal treatment discomfort, 16/20 at 1st laser treatment; 17/20 at 2nd laser Rx and 20/20 at 3rd patients recorded no discomfort following laser. | 1 month after first session, 3 and 6 months after last treatment | N/A |
| Goldstein 2015 | Human Fibroblast Lysate Cream (HFLC) vs placebo cream | 12 weeks, 1g to affected area BD OR placebo cream, 1g to affected area BD for 12 weeks. | N/A | VAS PR: HFLC cream= 43% decrease in pruritus (p=0.005) vs 51% decrease on placebo (p=0.001). No between group difference (p=0.226). VAS BP = 51% reduction in vulvar burning and pain in HFLC cream (p=0.002), 43% reduction in placebo (p=0.005). No between group difference (p=0.86) | Female Sexual Function Index - no difference from baseline in either group. | Investigator Global Assessment (IGA) (lichenification, ulceration, and induration) 0–3 scale: improvement with HFLC and placebo compared to baseline, no between group difference. mean decrease in IGA points was 0.9 (2.6–1.7) for the HFLC group and 0.5 (2.3–1.8) for placebo group, not statistically significant (p > 0.2). | N/A | Change in inflammation on biopsy - no change with placebo or HFLC cream. | No adverse events recorded during trial. | screening week -2, baseline week 0, follow-up week 12 |  |
| Goldstein 2019 | Autologous platelet-rich plasma intradermal injections vs placebo saline injection | 2 treatments, 6 weeks apart. 5ml intradermal and subdermal injections OR placebo saline injections 5ml twice separated by 6 weeks. | N/A | Clinical Scoring System for Vulvar Lichen Sclerosus (CSS) (patient domain) . Mean difference -7.74 PRP vs -9.44 control, no significant diff (Mann-Whitney U test result 80.50 [P = .654] | N/A | N/A | N/A | Change in inflammation on biopsy - PRP 5/19 improved, 10/19 no change, 4/19 worse. Control 5/10 improved, 4/10 no change, 1/10 worse. No difference between groups p=0.542 | adverse events - bruising, rate not reported. | N/A | N/A |
