# Supplementary material for: A systematic review of outcome measures evaluating treatment efficacy in vulval lichen sclerosus and evaluation of patients' priorities
Source: Skin Health Dis. 2024 Jul 5;4(5):e422. doi: 10.1002/ski2.422 (PMC11442075; doi:10.1002/ski2.422)
Supplement: Supplementary file 4 — Table S2 [file SKI2-4-e422-s004.docx]

| **Author and Year**  Table 2: Outcome Measures Reported in the included studies. | **Therapeutic and control interventions** | **Patient rated outcomes: Symptoms** | **Patient rated QOL outcomes** | **Physician rated sign-based outcomes** | **Objective: histology** | **Other outcomes** |
| --- | --- | --- | --- | --- | --- | --- |
| Paslin 1991 | Topical dihydrotestosterone 2% vs petrolatum ointment | The severity of itching and dyspareunia, qualitative reporting; no scale used. | N/A | Clinical photography and clinical assessment of hyperkeratosis, softening, loosening of vulval skin, and clitoral enlargement. | Histological evaluation of a change in elastin production. | N/A |
| Cattaneo 1996 | Topical testosterone propionate 2% vs petrolatum ointment | The severity of symptoms including itching; no scale used. | N/A | N/A | N/A | N/A |
| Paslin 1996 | Topical dihydrotestosterone 2% vs topical testosterone propionate 2% | 4-point scale was to assess itching and dyspareunia (0=none, 1 = mild, 2=moderate, 4=severe) recorded in a symptom diary. | Qualitative patient-reported change in sexual activity, bleeding, and ability to orgasm. | Clinical photography and clinical assessment of hyperkeratosis, fissures, and overall vulval skin appearance. | Histological microscopic evaluation of elastin fibre formation and composition of epithelium. | N/A |
| Sideri 1994 | Topical testosterone propionate 2% vs petrolatum ointment | Change in symptom severity expressed as a 3-level scale: Improved, Unchanged and Worsened; symptoms evaluated not further specified. | N/A | N/A | Histological assessment of therapeutic response; features evaluated not further specified. | N/A |
| Goldstein 2011 | Topical pimecrolimus 1% vs topical clobetasol propionate 0.05% | Patient-reported change in pruritus severity assessed with a 0-10-point visual analogue scale (VAS-PR) and change in burning and pain assessed with a 0-10-point visual analogue scale (VAS BP). | N/A | Clinical evaluation of an Investigator Global Assessment (IGA) of the severity of disease (0-3 scale), clinical evaluation of lichenification (0-3 scale), and clinical evaluation of ulceration/fissuring (0-3 scale). Assessment based on clinical photography and clinical examination. | Biopsy samples evaluated by dermatopathologist to evaluate the change in inflammation. | N/A |
| Origoni 1996 | Topical oxatomide gel 5% vs petrolatum ointment | Patient-reported severity of itching, burning and dyspareunia; graded as absent, mild, moderate, severe. | N/A | Clinician-assessed score-based scale was used to evaluate clinical appearance | N/A | Clinician-assessed score-based scale also included adverse side effects and local tolerability. |
| Burrows 2011 | Topical pimecrolimus 1% vs topical clobetasol propionate 0.05% | N/A | Female Sexual Distress Scale | N/A | Biopsy samples | N/A |
| D'Antuono 2011 | Dermasilk briefs vs cotton underwear | Subjective symptoms (burning, dryness, soreness, dyspareunia, itching, urinary problems, constipation) scored 0-3. | N/A | Objective genital signs (erythema, atrophy, fissures, whitening, vaginal stenosis, telangiectasia, erosions, purpura, hyperpigmentation, bleeding) | N/A | N/A |
| Gunthert 2022 | Topical progesterone cream 8% vs topical clobetasol propionate 0.05% | Lichen sclerosus symptom severity score consisting of Short Form SF-12 physical and mental health scores, and patient-reported symptom score of pruritus, itching, burning. | N/A | Clinical severity score for Lichen Sclerosus assessing erosion, hyperkeratosis, fissures, agglutinations, stenosis, atrophy. | Biopsy samples to assess change in inflammation | N/A |
| Virgili 2014 | Topical clobetasol propionate ointment 0.05% vs topical mometasone furoate 0.1% ointment | Visual Analogue Scale (VAS) to evaluate itching and burning (0-10 scale for each symptom). Global subjective score (GSS =20) obtained by adding score for itching and burning. Treatment responsive if a score ≤ 3 for both itching and burning. | N/A | Four-point scale (0=absence, 1=mild, 2=moderate, 3=severe) used to assess erythema, leukoderma, hyperkeratosis and purpuric lesions and itching-related excoriations. Global objective score (GOS) was obtained by adding scores for each clinical parameter (highest GOS=12). Assessed by a clinician. Treatment responsive if GOS ≤4. | N/A | Patients-reported treatment satisfaction and degree of convenience. |
| Borghi 2015 | Topical mometasone furoate 0.1% continuous application vs topical mometasone furoate 0.1% tapering dose | Visual Analogue Scale (VAS) to evaluate itching and burning (0-10 scale for each symptom). Global subjective score (GSS =20) obtained by adding score for itching and burning. Treatment responsive if a score ≤ 3 for both itching and burning. | N/A | Four-point scale (0=absence, 1=mild, 2=moderate, 3=severe) used to assess erythema, leukoderma, hyperkeratosis and purpuric lesions and itching-related excoriations. Global objective score (GOS) was obtained by adding scores for each clinical parameter (highest GOS=12). | N/A | N/A |
| Corazza 2016 | Topical mometasone furoate 0.1% vs topical clobetasol propionate 0.05% | Visual Analogue Scale (VAS) to evaluate itching and burning (0-10 scale for each symptom). Global subjective score (GSS =20) obtained by adding score for itching and burning. Treatment responsive if a score ≤ 3 for both itching and burning. | N/A | Four-point scale (0=absence, 1=mild, 2=moderate, 3=severe) used to assess erythema, leukoderma, hyperkeratosis and purpuric lesions and itching-related excoriations. Global objective score (GOS) was obtained by adding scores for each clinical parameter (highest GOS=12). | N/A | Patient-reported satisfaction, degree of convenience and adherence. Relapse rate. |
| Virgili 2013 | Topical mometasone furoate 0.1% vs topical vitamin E cream or cold cream | Visual analogue scale of burning, itching and dyspareunia obtained by interview. Score of 0-10 allocated to each symptom and a Global Subjective Score was obtained by adding individual scores (highest score=30). | N/A | Four-point scale (0=absence, 1=mild, 2=moderate, 3=severe) used to assess erythema, leukoderma, hyperkeratosis and purpuric lesions and itching-related excoriations. Global objective score (GOS) was obtained by adding scores for each clinical parameter (highest GOS=12). Treatment responsive if GOS ≤4. | N/A | Patients-reported treatment satisfaction and degree of convenience. One year relapse rate defined by a score > 5 for at least one evaluable subjective symptom and/or a score = 3 for any of the four signs considered reversible. The median time to relapse. |
| Shi 2016 | 5-Aminolevulinic Acid Photodynamic Therapy vs Topical Clobetasol Propionate 0.05% | Horizontal Visual Analogue Scale used to assess pruritus, burning and pain. Four level scale where 0= absent, 1=mild, 2= moderate, 3= severe. | N/A | Horizontal Visual Analogue scale to assess the lesion size and scale. Four level scale where 0= absent, 1= mild, 2= moderate, 3 = severe. Assessed using clinical photographs. | N/A | N/A |
| Burkett 2021 | Fractionated Carbon Dioxide Laser vs topical clobetasol propionate 0.05% | Validated visual analogue scale (subjective VAS) including the following symptoms: vulvar itching, vulvar burning, vulvar irritation, pain with intercourse, tearing of the vulvar skin, painful urination, and painful defecation from 0-10). Vulvovaginal Symptoms Questionnaire. Patient Global Impression of Improvement (PGI-I) on 0-5 VAS. | Skindex-29 questionnaire | Objective VAS: visual appearance, including white plaques or hypopigmentation, cigarette paper or thin skin, introital narrowing, perianal involvement (figure-of eight shape), loss of labia minora, fusion of labia minora, phimosis of clitoral hood, vulvar fissure, and erosion, scaled on a validated VAS from 0 to 10. Vaginal Health Index (VHI). | N/A | N/A |
| Gutierrez-Ontalvilla 2022 | Lipofilling and Platelet-Rich Plasma vs topical clobetasol propionate 0.05% | Global Subjective Score (GSS) was obtained by adding individual patient-reported scores for pruritus, burning sensation, pain, and dyspareunia. Each parameter was graded on a 11-point Visual Analogue Scale (0=no symptoms, 10=severe symptoms). GSS score ranged from 0-40. | Skindex-29 questionnaire | Global objective score (GOS) was obtained by adding the scores for seven clinical parameters (erosions, agglutination, hyperkeratosis, stenosis, fissures, atrophy, and leukoderma) assessed by a clinician, GOS range was 0-14. Each sign was graded on a 3-point Likert scale (0=normal findings, 1= moderate alterations, 2=severe changes). | Histopathological assessment by a blinded anatomopathologist. | Skin elasticity was determined using Cutometer Skin Elasticity Meter MPA 580 Dual - clitoral hood. |
| Funaro 2014 | Topical tacrolimus 0.1% vs topical clobetasol propionate 0.05% | Patient-reported symptoms; 0-10 scale of anogenital burning pain (VAS-BP) and pruritus (VAS-PR). Patient-reported change in condition, graded as worse, no difference, slightly better, moderately better, significantly better, or complete remission (6-point scale). | N/A | Clinician-assessed reduction in signs including white papules and/or patches, atrophy, erosion and ulcerated lesions, erythematous patches, lichenification in 5 regions of the anogenital area: perianal, perineal, labia majora, labia minora, and the clitoris) Scored 0-3. | N/A | N/A |
| Mitchell 2021 | Fractionated Carbon Dioxide Laser vs sham laser | Clinical Scoring System (CSS) for Vulvar Lichen Sclerosus, patient-reported component; 0-10 VAS scale to evaluate four domains: pruritus, soreness, burning and dyspareunia. Subjective CSS scored from 0 to 40. | N/A | Clinical Scoring System (CSS) for Vulvar Lichen Sclerosus, clinician's objective section scored from 0 to 12, a sum of six domains: fissures, erosions, hyperkeratosis, agglutination, stenosis, and atrophy. | Histopathology severity scale, 7 point (0-6), evaluated by a gynaecological pathologist. Scale quantifies the loss of rete pegs, the amount of dermal homogenization, the amount of chronic inflammation. | N/A |
| Bijzak Ogrnic 2019 | Nd:YAG laser 1064nm vs topical betamethasone | VAS scores sum of Burning, itching and pain. | Questionnaire on sexual activity, lack of sensation during intercourse, anorgasmia, and dyspareunia. | Evaluation of photographs to assess a change in signs and assign an improvement score graded as 0=no improvement, 1=poor improvement, 2=partial improvement, 3=complete improvement). If assessors assigned the order of photographs incorrectly, their score was given a negative value. | Vulva punch biopsies to assess thickness of epidermis and sclerosis with assessment of inflammation on 4-point scale (none, mild, moderate, severe). | Patient-reported satisfaction with treatment graded as 0=very unsatisfied; 1=unsatisfied; 2=satisfied; 3=very satisfied). Tolerability of laser evaluated on a 0-10 VAS scale. |
| Goldstein 2015 | Human Fibroblast Lysate Cream vs placebo cream | VAS PR (Pruritus, vulvar burning, pain) 0-10 scale. | Female Sexual Function Index | Investigator Global Assessment (IGA) evaluating lichenification, ulceration and induration on a 0-3 scale. Assessment with clinical photographs. | Change in inflammation on biopsy; no objective scale used. | N/A |
| Goldstein 2019 | Autologous platelet-rich plasma intradermal injections vs placebo saline injection | Clinical Scoring System (CSS) for Vulvar Lichen Sclerosus, patient-reported component; 0-10 VAS scale to evaluate four domains: pruritus, soreness, burning and dyspareunia. Subjective CSS scored from 0 to 40. | N/A | N/A | Blinded rating of inflammatory infiltration on the pre-treatment and post-treatment biopsy specimens (on a 0-3 scale). | N/A |

Table 2 (continued): Outcome Measures Reported in Randomised Controlled Trials
